# Mid-term results of surgical aortic valve replacement with bioprostheses in hemodialysis patients

**DOI:** 10.1016/j.ijcha.2022.101030

**Published:** 2022-04-11

**Authors:** Ikuko Shibasaki, Taira Fukuda, Hironaga Ogawa, Go Tsuchiya, Yusuke Takei, Masahiro Seki, Takashi Kato, Yuta Kanazawa, Shunsuke Saito, Toshiyuki Kuwata, Yasuyuki Yamada, Yasuo Haruyama, Hirotsugu Fukuda

**Affiliations:** a Department of Cardiac and Vascular Surgery, Dokkyo Medical University, Tochigi, Japan; b Department of Liberal Arts and Human Development, Kanagawa University of Human Services, Kanagawa 238-8522, Japan; c Division of Cardiovascular Surgery, Gunma Prefectural Cardiovascular Centre, Gunma, Japan; d Department of Cardiovascular Surgery, Maebashi Red Cross Hospital, Gunma, Japan; e Center for Research Collaboration and Support, Dokkyo Medical University School of Medicine, Japan

**Keywords:** Hemodialysis, Surgical aortic valve replacement, Bioprostheses, Aortic valve stenosis, HD, hemodialysis, HF, heart failure, MI, myocardial infarction, AS, aortic stenosis, SAVR, surgical aortic valve replacement, TAVR, transcatheter aortic valve replacement, SAVR-BP, SAVR with bioprostheses, AXA, axillary artery, CPB, cardiopulmonary bypass, CP, cold blood cardioplegia, ICU, intensive care unit, CABG, coronary artery bypass graft, APACHE, Acute Physiology and Chronic Health Evaluation, SOFA, Sequential Organ Failure Assessment, SAPS II, Simple Acute Physiology Score II, SD, standard deviation, ECMO, extracorporeal membrane oxygenation, Af, atrial fibrillation, MACCE, major adverse cardiovascular and cerebrovascular events, LV, left ventricular, Dd, diastolic diameter, Ds, systolic diameter, IABP, intra-aortic balloon pumping, LVEF, left ventricle ejection fraction, 95% CI, confidence interval, HR, hazard ratio, LOS, low output syndrome

## Abstract

**Background:**

Limited studies have assessed the factors affecting prognosis in hemodialysis (HD) patients who undergo surgical aortic valve replacement with a bioprostheses (SAVR-BP). This study aimed to evaluate the outcomes of HD patients who had undergone SAVR-BP for aortic stenosis (AS) and identify the risk factors for mortality.

**Methods:**

This retrospective study included 57 HD patients who had undergone SAVR-BP for AS between July 2009 and December 2020. Multivariate logistic regression was used to predict factors associated with mid-term outcomes and death or survival. Kaplan − Meier curves were also generated for mid-term survival.

**Results:**

The in-hospital mortality rate was 8.8%, and the 5-year mortality rate was 42.1%. The independent predictors of 5-year mortality were preoperative age (hazard ratio [HR], 1.57; 95% confidence interval [CI], 1.175–2.083, p = 0.002), hyperlipidemia (HR, 0.02; 95% CI, 0.002–0.297, p = 0.004), left ventricular diastolic diameter (HR, 1.74; 95% CI, 1.142–2.649, p = 0.010), left ventricular systolic diameter (HR, 0.61; 95% CI, 0.392–0.939, p = 0.025), and Japan SCORE (HR, 1.28; 95% CI, 1.052–1.563, p = 0.014). The postoperative predictors included intensive care unit stay (HR, 1.11; 95% CI, 1.035–1.194, p = 0.004) and albumin level (HR, 0.38; 95% CI, 0.196–0.725, p = 0.003).

**Conclusions:**

The 5-year prognosis of HD patients undergoing SAVR may be improved by early diagnosis (before the occurrence of LV hypertrophy/enlargement) and nutritional management with oral intake to alleviate postoperative hypoalbuminemia.

**Registration number of clinical studies:** UMIN000047410.

## Highlights

• HD patients underwent SAVR-BP for AS (hospital mortality, 8.8%; 5-year mortality, 42.1%).
• Preoperative risk factors for 5-year mortality: age, hyperlipidemia, LVDd, LVDs, and Japan SCORE.
• Postoperative risk factors for 5-year mortality: length of ICU stay, and albumin level at discharge.

## 1 Introduction

In recent years, the number of new patients undergoing hemodialysis (HD) has been increasing, primarily because of the aging population and the evolution of HD techniques [1]. The number of annual deaths has also been increasing. According to a report by the Japanese Society for HD therapy, heart failure (HF) was the most common cause of death in 2019 (22.7%), followed by infections (21.5%), malignancies (8.7%), cerebrovascular disease (5.7%), and myocardial infarction (MI) (3.9%) [2]. The frequencies of calcification associated with intimal atherosclerosis and tunica media are high in HD patients [3], and atherosclerosis and calcification are risk factors for cardiovascular disease [4], [5]. Moreover, ectopic calcification reportedly progresses more rapidly in HD patients than it does in non-HD patients [6], [7]. The incidence of aortic stenosis (AS) in HD patients has been increasing annually and is a leading cause of death. The standard treatment for AS is surgical aortic valve replacement (SAVR); however, transcatheter aortic valve replacement (TAVR) has recently been approved in Japan. Maeda et al. [8] reported that the 3-year overall survival rate of TAVR in HD patients with AS was 55.7%, and 12% had valve dysfunction, which raised the issue of complications and valve durability specific to HD patients. In SAVR, surgical mortality and morbidity rates are also higher in HD patients than in non-HD patients [9], [10], [11]. Few studies have assessed the prognostic determinants of the disease. Elucidating the prognostic factors is important to improve the prognosis of SAVR in HD patients. Therefore, in this study, we investigated the preoperative and postoperative risk factors for mid-term mortality following SAVR with bioprostheses (SAVR-BP) in HD patients.

## 2 Methods

### 2.1 Study population

Between July 2009 and December 2020, 703 patients underwent SAVR for AS at our institution. Out of 61 (8.7%) patients who underwent HD, we included 57 (8.1%) who underwent SAVR-BP. We divided the 57 patients into two groups based on the 5-year mortality rates (33 patients in the survival group and 24 patients in the non-survival group) and investigated factors affecting mortality. The selection criterion for bioprosthetic valves was age > 60 years, regardless of whether the patient was receiving HD. The study protocol was approved by the Dokkyo Medical University Hospital Ethics Committee (Approval No: R-49–15 J) and the requirement for informed consent was waived.

### 2.2 Surgical management

Preoperative contrast-enhanced computed tomography and intraoperative epiaortic ultrasound were performed to confirm the clamping and cannulation sites. The right axillary artery (AXA) was selected when cannulation was not possible. In cases where aortic clamping was difficult due to severe calcification, ascending aortic replacement under hypothermic circulatory arrest was used as a combined procedure. All surgeries were performed through a complete median sternotomy. A cardiopulmonary bypass (CPB) was placed between the aorta or the right AXA and the right atrium, and myocardial protection was provided using high-potassium cold blood cardioplegia (CP). CP was administered in the following order: antegrade, retrograde, and selectively, followed by retrograde, every 15 min. After surgery, all patients were transferred to the intensive care unit (ICU). The ventilator was removed when the patient was hemodynamically stable, had no postoperative bleeding, and was fully conscious. All patients received regular HD the day before surgery, and HD was resumed on the first postoperative day. Continuous hemodiafiltration was used if the patient was hemodynamically unstable.

### 2.3 Evaluation of the severity of illness and organ damage index in emergency intensive care

We used the Japan SCORE data from the Japanese Adult Cardiovascular Surgery Database in a risk analysis model. We calculated the predicted operative mortality (30-day mortality + in-hospital mortality) and 30-day operative mortality + major complications by assessing the preoperative clinical data and procedure-related patient information (single coronary artery bypass graft (CABG), valve surgery, and aortic surgery). The database reflects the clinical outcomes of cardiovascular surgery in Japan and is, therefore, considered to be close to the actual scenario [12], [13], [14]. In addition to the Japan SCORE, the Acute Physiology and Chronic Health Evaluation (APACHE) II score, Sequential Organ Failure Assessment (SOFA) score, and Simple Acute Physiology Score II (SAPS II) were used in this study. These are prognostic methods designed to objectively evaluate the severity of illness in patients admitted to the ICU [15]. The APACHE II score is calculated from physiological parameters (the most abnormal value for each of the 12 parameters measured), age modification, and assessment of chronic disease obtained within 24 h of admission to the ICU [16]. The SOFA score is a mortality prediction score that is based on the degree of dysfunction in the six organ systems (that is, respiration, blood coagulation, and the liver, circulatory system, central nervous system, and kidney). The parameters include PaO_2_/FiO_2_ (mmHg), platelet count (×103/mm^2^), bilirubin level (mg/dL), hypotension, Glasgow Coma Scale, creatinine level (mg/dL), or urine output, which are evaluated on a 5-point scale. The score is calculated on admission and every 24 h until discharge, using the worst parameters measured during the previous 24 h [17]. The SAPS II score is a simpler version of the APACHE II score, scored using 17 items: 12 physiological variables, age, mode of admission, and three underlying diseases (AIDS, hematologic diseases, and metastatic malignancies) [18].

### 2.4 Statistical analysis

Continuous variables are presented as mean and standard deviation (SD), and categorical variables are presented as counts and proportions. The 5-year survival was calculated using the Kaplan − Meier analysis. Comparison of the 5-year outcomes was performed using the chi-square test for categorical variables and the Mann − Whitney *U* test for continuous variables, as appropriate. All variables with p-values < 0.2 were categorized as univariate predictors. A multivariate analysis using Cox proportional hazards model was performed to identify the independent predictors for 5-year mortality. In the multivariate analysis, we categorized the variables into preoperative, intraoperative, and postoperative variables. A two-sided p-value < 0.05 was considered statistically significant. IBM SPSS Statistics software version 27.0 (IBM Corp., Armonk, NY, USA) was used for the statistical analyses.

## 3 Results

### 3.1 Preoperative and intraoperative characteristics

Table 1 summarizes the baseline characteristics of the study population. The mean age of the study population was 73.5 years (SD 7.3 years), and 35 (61.4%) participants were men. Patients were very likely to have a history of hypertension (89.5%), hyperlipidemia (49.1%), and diabetes mellitus (54.4%). The mean pressure gradient was 50.4 mmHg (SD 14.7 mmHg), and the mean aortic valve area was 0.6 cm^2^ (SD 0.2 cm^2^). The mean brain natriuretic peptide levels were high at 2526.7 pg/mL (SD 2434.3 pg/mL). Emergency surgery was performed in 11 patients (19.3%), and the mean time for CPB was 180.2 min (SD 41.0 min). Concomitant surgery was performed in 36 patients (63.2%), with tricuspid valvuloplasty being the most common (25 [43.9%] patients), followed by CABG (12 [21.1%] patients). All valves used were stented, with bovine pericardial valves in 56 cases and porcine valves in one case. The valve sizes used were 19, 21, and 23 mm in 18, 26, and 13 patients, respectively. The mean transfusion of red blood cells (RBC) was 10.5 units (SD 8.2 units). The mean SOFA, APACHE II, and SAPS scores after admission to the ICU were 10.9 (SD 2.6), 24.0 SD (6.0), and 49.9 (SD 11.1), respectively.

**Table 1:** Pre- and postoperative characteristics of hemodialysis patients undergoing aortic valve replacement with biological valves.

	Preoperative	Postoperative
Variables (n = 57)	n (%)/mean [SD]	n (%)/mean [SD]
Age (years)	73.5 [7.3]	
Sex, male	35 (61.4)	
BSA (m^2^)	1.49 [0.19]	
Dialysis history (years)	11.7 [8.0]	
Peripheral arterial disease	19 (33.3)	
Hypertension	51 (89.5)	
Hyperlipidemia	28 (49.1)	
DM	31 (54.4)	
NYHA class	2.3 [0.8]	
Coronary artery disease	15 (26.3)	
Emergency surgery	11 (19.3)	
Operation time (min)	346.2 [119.9]	
CPB time (min)	182.0 [41.0]	
Aorta cross clamp time (min)	80.5 [34.6]	
Concomitant surgery CABG	12 (21.1)	
Valve type		
Stented bovine pericardial valve	56 (98.2)	
Stented porcine valve	1 (1.8)	
Size of valve 19 mm	18 (31.6)	
21 mm	26 (45.6)	
23 mm	13 (22.8)	
Transfusion of red blood cells (units)	10.5 [8.2]	
Japan SCORE	14.9 [16.2]	
Japan SCORE + major complications	30.4 [14.6]	
Electrocardiogram^a^		
Af	10 (18.5)	10 (17.5)
Echocardiographic variables		
LV IVST^a^ (mm)	11.6 [2.2]	10.4 [2.2]
LV PWth^a^ (mm)	11.0 [1.8]	10.3 [1.7]
LV diastolic diameter^a^ (mm)	48.5 [6.5]	44.2 [10.3]
LV systolic diameter^a^ (mm)	31.5 [7.6]	30.0 [8.5]
LV ejection fraction^a^ (%)	54.7 [20.5]	58.7 [10.8]
Mean PG^a^ (mmHg)	50.4 [14.7]	11.8 [6.0]
Aortic valve area^a^ (cm^2^)	0.6 [0.2]	1.5 [0.4]
Blood test		
Albumin (g/dL)	3.6 [0.5]	2.8 [0.6]
T-cho^a^ (mg/dL)	154.0 [32.2]	
Triglycerid^a^ (mg/dL)	88.1 [31.4]	
HDL cho^a^ (mg/dL)	52.6 [13.6]	
LDL cho^a^ (mg/dL)	85.4 [26.0]	
Hemoglobin (g/dL)	12.0 [2.0]	9.9 [1.5]
BNP^a^ (pg/mL)	2,526.7 [2,434.3]	736.1 [532.4]
IABP	5 (8.8)	5 (8.8)
ECMO	3 (5.3)	4 (7.0)
SOFA score		10.9 [2.6]
APACHE II score		24.0 [6.0]
SAPS II score		49.9 [11.1]
Intubation time (h)		78.6 [133.7]
ICU stay (days)		3.6 [4.7]

SD, standard deviation; BSA, body surface area; DM, diabetes mellitus; NYHA, New York Heart Association; Af, atrial fibrillation; LV, left ventricular; PG, pressure gradient; BNP, brain natriuretic peptide; IABP, intra-aortic balloon pumping; ECMO, extracorporeal membrane oxygenation; CPB, cardiopulmonary bypass; CABG, coronary artery bypass grafting; SOFA, sequential organ failure assessment; APACHE, acute physiology and chronic health evaluation; SAPS, simplified acute physiology score; ICU, intensive care unit; LV IVST, left ventricular interventricular septal thickness; LV PWth, left ventricular posterior wall thickness

a: Using the Chi-squared test or Mann–Whitney *U* test.

b: Missing values are excluded (preoperative electrocardiogram = 3, LV IVST = 14, LV PWth = 15, LV diastolic diameter = 4, LV systolic diameter = 4, LV ejection fraction = 1, mean PG = 10, aortic valve area = 2, BNP = 1, postoperative electrocardiogram = 1, LV IVST = 3, LV PWth = 1, LV diastolic diameter = 5, LV systolic diameter = 6, LV ejection fraction = 3, mean PG = 7, aortic valve area = 9, and BNP = 18).

### 3.2 Early outcomes

In-hospital mortality was observed in five patients (8.8%). The causes of hospital death included low output syndrome (LOS) (n = 2 [40%]), sepsis (n = 2 [40%]), and non-occlusive mesenteric ischemia (NOMI) (n = 1 [20%]). The most common major complication was prolonged ventilation (n = 7 [12.3%]), followed by deep sternal infection (n = 6 [10.5%]), septicemia (n = 4 [7.0%]), stroke (n = 3 [5.3%]), pneumonia (n = 2 [3.5%]), gastrointestinal complications (n = 3 [5.3%]), and perioperative MI (n = 1 [40.6%]) (Table 2).

**Table 2:** Mortality and morbidity of hemodialysis patients who underwent aortic valve replacement with biologic valves.

Parameter	n = 57
Mortality	
In-hospital mortality	5 (8.8)
LOS	2 (3.5)
Pneumonia	2 (3.5)
Septicemia (NOMI)	1 (1.6)
1-year	5 (8.8)
Complications	
Deep sternum infection	6 (10.5)
Septicemia	4 (7.0)
Stroke	3 (5.3)
Pneumonia	2 (3.5)
Gastrointestinal complications	3 (5.3)
Perioperative MI	1 (1.6)

Data are presented as numbers (%).

LOS, low output syndrome; NOMI, non-occlusive mesenteric ischemia;

MI, myocardial infarction

### 3.3 Five-year mortality outcomes

**Fig. 1:**
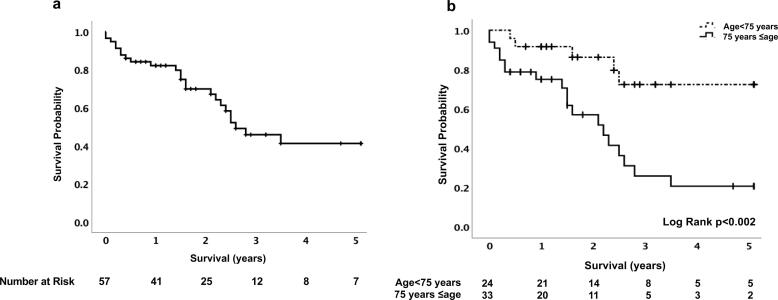
Kaplan–Meier survival curve of 5-year mortality after bioprosthetic aortic valve replacement for aortic stenosis. **a:** hemodialysis patients. **b:** comparison of the < 75 years age group vs. ≥ 75 years age group (long-rank test).

The 5-year cumulative survival rate was 82.1% at 1 year, 45.9% at 3 years, and 41.3% at 5 years (Fig. 1). Furthermore, we compared survival rates at a median age of 75 years in two groups, including the < 75 years age group and the ≥ 75 years age group, and observed that the survival rate was significantly higher in the < 75 years age group than in the ≥ 75 years age group (p < 0.002). There were 24 patients (42.1%) with postoperative 5-year mortality, and the causes of death were septicemia (n = 5 [20.8%]), LOS (n = 4 [16.7%]), multi-organ failure (n = 2 [8.3%]), ventricular fibrillation (n = 2 [8.3%]), brain complications (n = 2 [8.3%]), MI (n = 1 [4.2%]), gastrointestinal bleeding (n = 1 [4.2%]), lung cancer (n = 1 [4.2%]), and NOMI (n = 1 [4.2%]), with unknown causes in six patients. During this period, major adverse cardiovascular and cerebrovascular events (MACCE) occurred in seven patients (12.3%), and prosthetic valve endocarditis occurred in three patients (5.3%); there was no structural valve deterioration. The non-survival group (n = 24) showed significant differences in the following variables compared to the survival group (n = 33). Especially, preoperatively, non-survival patients were older, had a lower incidence of hyperlipidemia, and required extracorporeal membrane oxygenation (ECMO). Intraoperatively, RBC use was higher in non-survival patients (Table 3). Postoperatively, non-survival patients were more likely to require ECMO. The SOFA, APACHE II, and SAPS II scores at ICU admission were higher in the non-survival than in the survival group; however, only the SOFA score was significantly different. Non-survival patients were more likely to have atrial fibrillation (Af) and hypoalbuminemia at discharge, and postoperative septicemia (Table 4).

**Table 3:** Comparison of preoperative and intraoperative factors associated with 5-year mortality in hemodialysis patients undergoing bioprosthetic aortic valve replacement.

	Survivor group (n = 33)	Non-survivor group (n = 24)	
Variables	n (%)/mean [SD]	n (%)/mean [SD]	p-value^a^
Age (years)	72.0 [7.4]	75.6 [6.8]	**0.031**
Sex, male	18 (54.5)	17 (70.8)	0.212
BSA (m^2^)	1.50 [0.22]	1.47 [0.16]	0.550
Dialysis history (years)	10.9 [8.0]	9.1 [7.7]	0.315
Peripheral arterial disease	13 (39.4)	6 (25.0)	0.255
Hypertension	31 (93.9)	20 (83.3)	0.198
Hyperlipidemia	20 (60.6)	8 (33.3)	**0.042**
DM	19 (57.6)	12 (50.0)	0.571
NYHA class	2.4 [0.8]	2.2 [0.9]	0.479
Coronary artery disease	10 (30.3)	5 (20.8)	0.794
Emergency surgery	5 (15.2)	6 (25.0)	0.802
Operation time (min)	341.2 [123.9]	353.2 [116.4]	0.698
CPB time (min)	180.7 [79.6]	190.5 [59.2]	0.341
Aorta cross clamp time (min)	141.0 [70.4]	138.5 [51.5]	0.862
Concomitant surgery CABG	8 (24.2)	4 (16.7)	0.489
Transfusion of red blood cells (unit)	8.8 [4.6]	13.0 [8.2]	**0.035**
Japan SCORE	11.9 [10.9]	18.1 [19.9]	0.133
Electrocardiogram^b^			
Af	3 (9.7)	7 (30.4)	0.052
Echocardiographic variables			
LV IVST^b^ (mm)	11.5 [2.3]	11.9 [2.0]	0.592
LV PWth^b^ (mm)	11.1 [1.8]	10.8 [1.9]	0.885
LV diastolic diameter^b^ (mm)	48.2 [7.2]	51.3 [10.8]	0.120
LV systolic diameter^b^ (mm)	32.0 [8.7]	35.9 [11.8]	0.102
LV ejection fraction^b^ (%)	58.4 [15.7]	55.7 [16.4]	0.446
Mean PG^b^ (mmHg)	52.0 [12.8]	50.7 [22.8]	0.763
Aortic valve area^b^ (cm^2^)	0.67 [0.20]	0.64 [0.17]	0.633
Blood test			
Albumin (g/dL)	3.4 [0.5]	3.3 [0.4]	0.501
T-cho^b^ (mg/dL)	160.3 [29.8]	149.0 [35.0]	0.083
Triglycerid^b^ (mg/dL)	101.3 [46.5]	90.8 [43.2]	0.351
HDL cho^b^ (mg/dL)	51.7 [14.0]	51.0 [11.3]	0.943
LDL cho^b^ (mg/dL)	89.9 [29.0]	82.2 [29.0]	0.181
Hemoglobin (g/dL)	11.5 [1.9]	10.7 [1.4]	0.169
BNP^b^ (pg/mL)	1661.4 [2159.9]	2564.3 [2834.5]	0.136
IABP	2 (6.1)	3 (12.5)	0.396
ECMO	0 (0)	3 (12.5)	**0.037**

SD, standard deviation; BSA, body surface area; DM, diabetes mellitus; NYHA, New York Heart Association; Af, Atrial fibrillation; T-cho, total cholesterol; HDL, high-density lipoprotein; LDL, low-density lipoprotein; BNP, brain natriuretic peptide; LV, left ventricular; IVST, interventricular septum thickness; PWth, posterior wall thickness; PG, pressure gradient; IABP, intra-aortic balloon pumping; ECMO, extracorporeal membrane oxygenation; CPB, cardiopulmonary bypass; CABG, coronary artery bypass grafting; SOFA, sequential organ failure assessment; APACHE, acute physiology and chronic health evaluation; SAPS, simplified acute physiology score; ICU, intensive care unit

a: Using the Chi-squared test or Mann-Whitney *U* test.

b: Missing values were excluded (electrocardiogram = 3, LV IVST = 14, LV PWth = 15, LV diastolic diameter = 4, LV systolic diameter = 4, LV ejection fraction = 1, mean PG = 10, aortic valve area = 2, T-cho = 4, triglycerid = 4, HDL cho = 4, LDL cho = 4, and BNP = 1).

**Table 4:** Comparison of postoperative factors associated with 5-year mortality in hemodialysis patients undergoing bioprosthetic aortic valve replacement.

	Survivor group (n = 33)	Non-survivor group (n = 24)	
Variables	n (%)/mean [SD]	n (%)/mean [SD]	p-value^a^
IABP	1 (3.0)	4 (16.7)	0.072
ECMO	0 (0)	4 (16.7)	**0.015**
SOFA score	10.0 [2.4]	12.0 [2.5]	**0.004**
APACHE II score	23.0 [4.6]	25.3 [7.5]	0.311
SAPS II score	48.6 [8.7]	51.5 [13.7]	0.616
Intubation time (hours)	29.7 [78.4]	73.0 [125.0]	0.421
ICU stay (days)	2.3 [3.3]	5.08 [8.3]	0.065
Electrocardiogram^b^			
Af	3 (9.1)	7 (29.2)	**0.049**
Echocardiographic variables			
LV IVST^b^ (mm)	10.1 [2.1]	10.8 [2.2]	0.402
LV PWth^b^ (mm)	10.3 [1.4]	10.3 [2.2]	0.985
LV diastolic diameter^b^ (mm)	45.3 [9.1]	42.4 [12.0]	0.707
LV systolic diameter^b^ (mm)	31.5 [7.8]	27.7 [9.5]	0.417
LV ejection fraction^b^ (%)	59.1 [10.2]	58.2 [11.6]	0.942
Mean PG^b^ (mmHg)	11.8 [7.3]	12.0 [4.7]	0.443
Aortic valve area^b^ (cm^2^)	1.57 [0.43]	1.45 [0.31]	0.358
Blood test			
Albumin (g/dL)	3.0 [0.4]	2.5 [0.7]	**0.004**
Hemoglobin (g/dL)	10.2 [1.2]	9.5 [1.7]	0.271
BNP^b^ (pg/mL)	761.4 [532.5]	667.8 [519.8]	0.555
Complications			
Septicemia	0 (0)	4 (16.7)	**0.015**
Stroke	2 (6.1)	1 (4.2)	0.752
Pneumonia	0 (0)	2 (8.3)	0.091
Deep sternum infection	3 (9.1)	3 (12.5)	0.679
Gastrointestinal complications	1 (3.0)	2 (8.3)	0.376
Perioperative MI	0 (0)	1 (4.2)	0.237

IABP, intra-aortic balloon pumping; ECMO, extracorporeal membrane oxygenation; SOFA, sequential organ failure assessment; APACHE, acute physiology and chronic health evaluation; SAPS, simplified acute physiology score; ICU, intensive care unit; Af, Atrial fibrillation; LV, left ventricular; PG, pressure gradient; IVST, interventricular septal thickness; PWth, posterior wall thickness, BNP, brain natriuretic peptide; MI, myocardial infarction

a: Using the Chi-squared test or Mann–Whitney *U* test.

b: Missing values were excluded (LV IVST = 3, LV PWth = 6, LV diastolic diameter = 5, LV systolic diameter = 6, LV ejection fraction = 3, mean PG = 7, aortic valve area = 9, and BNP = 18).

### 3.4 Univariate predictors of 5-year mortality

Univariate predictors of operative death are summarized in Table 5. Predictors included age (hazard ratio [HR], 1.07; 95% confidence interval [CI], 1.008–1.1137, p = 0.026), preoperative Af (HR, 3.42; 95% CI, 1.368–8.525, p = 0.008), preoperative left ventricular (LV) diastolic diameter (Dd) (HR, 1.06; 95% CI, 1.002–1.117, p = 0.044), preoperative LV systolic diameter (Ds) (HR, 1.06; 95% CI, 1.011–1.102, p = 0.015), Japan SCORE (HR, 1.03; 95% CI, 1.006–1.050, p = 0.014), preoperative ECMO (HR, 89.45; 95% CI, 8.987–890.300, p < 0.001), intraoperative RBC transfusion (HR, 1.08; 95% CI, 81.016–1.139, p = 0.012), postoperative intra-aortic balloon pumping (IABP) (HR, 3.27; 95% CI, 1.108–9.640, p = 0.032), SOFA score (HR, 1.17; 95% CI, 1.019–1.334, p = 0.026), ICU stay (HR, 1.11; 95% CI, 1.045–1.169, p < 0.001), postoperative albumin level (HR, 0.58; 95% CI, 0.379–0.781, p = 0.011), septicemia (HR, 25.10; 95% CI, 5.943–106.020, p < 0.001), and pneumonia (HR, 10.94; 95% CI, 2.179–54.903, p = 0.004).

**Table 5:** Factors influencing 5-year mortality in hemodialysis patients undergoing bioprosthetic aortic valve replacement.

Variables	Univariate analysis				
	HR	95 %CI			p-value^a^
Age	1.07	1.008	–	1.137	**0.026**
Sex, male	2.127	0.872	–	5.19	0.097
Hypertension	0.59	0.201	–	1.749	0.344
Hyperlipidemia	0.54	0.229	–	1.254	0.150
Af (preoperative)	3.42	1.368	–	8.525	**0.008**
LV diastolic diameter (preoperative)	1.06	1.002	–	1.117	**0.044**
LV systolic diameter (preoperative)	1.06	1.011	–	1.102	**0.015**
T-cho (preoperative)	0.99	0.971	–	1.007	0.213
LDL-cho (preoperative)	0.99	0.971	–	1.010	0.334
Hemoglobin (preoperative)	0.80	0.625	–	1.033	0.088
BNP (preoperative)	1.00	1.000	–	1.000	0.170
Japan SCORE	1.03	1.006	–	1.050	**0.014**
ECMO (preoperative)	89.45	8.987	–	890.300	**<0.001**
Transfusion of red blood cells	1.08	1.016	–	1.139	**0.012**
IABP (postoperative)	3.27	1.108	–	9.640	**0.032**
ECMO (postoperative)	1.00	0.009	–	117.363	1.000
SOFA score	1.17	1.019	–	1.334	**0.026**
ICU stay	1.11	1.045	–	1.169	**0.000**
Af (postoperative)	2.06	0.849	–	4.979	0.110
Albumin (postoperative)	0.58	0.379	–	0.781	**0.011**

HR, hazard ratio; 95 %CI, 95% confidence interval; Af, atrial fibrillation; T-cho, total cholesterol; LDL, low density lipoprotein; BNP, brain natriuretic peptide; ECMO, extracorporeal membrane oxygenation; IABP, intra-aortic balloon pumping; SOFA, sequential organ failure assessment; ICU, intensive care unit

a Using Cox proportional hazards and each variable with p < 0.2 (Table 3, Table 4), excluding sex.

### 3.5 Multivariate predictors of 5-year mortality

Multivariate predictors of operative death are summarized in Table 6. Pre- and intraoperative predictors included age (HR, 1.57; 95% CI, 1.175–2.083, p = 0.002), hyperlipidemia (HR, 0.02; 95% CI, 0.002–0.297, p = 0.004), LVDd (HR, 1.74; 95% CI, 1.142–2.649, p = 0.010), LVDs (HR, 0.61; 95% CI, 0.392–0.939, p = 0.025), and Japan SCORE (HR, 1.28; 95% CI, 1.052–1.563, p = 0.014). Postoperative predictors included ICU stay (HR, 1.11; 95% CI, 1.035–1.194, p = 0.004) and albumin level (HR, 0.38; 95% CI, 0.196–0.725, p = 0.003).

**Table 6:** Independent factors influencing 5-year mortality in hemodialysis patients undergoing bioprosthetic aortic valve replacement.

Variables	Multivariate analysis		
	HR	95 %CI	p-value^a^
Pre- and intraoperative model			
Age	1.57	1.175–2.083	**0.002**
Sex, male	10.48	0.896–122.543	0.061
Hyperlipidemia	0.02	0.002–0.297	**0.004**
Af	2.18	0.346–13.691	0.407
LV diastolic diameter	1.74	1.142–2.649	**0.010**
LV systolic diameter	0.61	0.392–0.939	**0.025**
Hemoglobin	0.62	0.270–1.403	0.249
BNP	1.00	1.000–1.001	0.077
Japan SCORE	1.28	1.052–1.563	**0.014**
Transfusion of red blood cells	1.06	0.841–1.342	0.611
Postoperative model			
Age	1.12	1.038–1.207	**0.003**
Sex, male	8.67	2.571–29.247	**0.000**
IABP	1.25	0.268–5.798	0.778
SOFA score	1.18	1.000–1.380	0.050
ICU stay	1.11	1.035–1.194	**0.004**
Af	1.62	0.518–5.035	0.408
Albumin	0.38	0.196–0.725	**0.003**

HR, hazard ratio; 95 %CI, 95% confidence interval; Af, Atrial fibrillation; BNP, brain natriuretic peptide; ECMO, extracorporeal membrane oxygenation; IABP, intra-aortic balloon pumping; SOFA, sequential organ failure assessment; ICU, intensive care unit

a Selected using Cox proportional hazards model and variables with p < 0.2 (Table 5), excluding sex (as confounder) and ECMO (survivors = 0), septicemia (survivors = 0), and pneumonia (survivors = 0).

## 4 Discussion

In this study, we investigated the preoperative and postoperative risk factors contributing to 5-year mortality following SAVR-BP in HD patients. There were several important findings. First, in the 5-year non-survival group, patients were older at the time of surgery and a higher proportion of them required ECMO preoperatively. Intraoperatively, a higher proportion of patients required ECMO and RBC transfusion. At discharge, there was a higher prevalence of Af and hypoalbuminemia. Second, the independent risk factors for 5-year mortality were age, hyperlipidemia, LVDd and LVDs, and Japan SCORE in the preoperative period, and ICU length of stay and hypoalbuminemia in the postoperative period.

During the study period, a total of 57 (8.1%) HD patients underwent SAVR-BP for AS. The in-hospital mortality rate in this study was 8.8%, which was similar to that reported in previous studies (∼10%) [9], [19], [20], [21], [22]. The hospital mortality rate for dialysis patients undergoing TAVR, a minimally invasive procedure, is reported to be approximately 8%, similar to that of SAVR [23], [24]. The 5-year mortality rate has been reported to be 39–72%; in this study, it was 42.1% [20], [21], [22].

### 4.1 Preoperative risk factors for 5-year mortality

We found that age, hyperlipidemia, LVDd, Ds, and Japan Score were strong independent risk factors for mortality. Yamauchi et al. [19] analyzed the risk factors for perioperative mortality in HD patients from the Japanese Adult Cardiovascular Surgery Database. Preoperative and intraoperative risk factors included age, concomitant CABG, NYHA class IV, liver dysfunction, left ventricle ejection fraction (LVEF) 30–60%, peripheral arterial disease (PAD), Af, and history of cardiac surgery. Aljohani et al. [9] reported the following risk factors: age > 75 years, male sex, PAD, liver dysfunction, concomitant surgery, and preoperative IABP or ECMO support. As valve calcification progresses with age, valve mobility is reduced, resulting in valve dysfunction. Aortic valves are particularly prone to calcification in HD patients [25]. Valve calcification is also an independent risk factor for all-cause mortality and death from cardiovascular disease [3].

The mean age of patients who died in the hospital was 78.4 years (SD 2.4 years), and the mean age of patients who died before 5 years was 75.6 years (SD 6.8 years): notably, these mean ages were significantly higher than those of patients in the survival group. Additionally, a comparison of survival rates between the two groups at a median age of 75 years showed that the survival rate was significantly higher in the < 75 years age group than in the ≥ 75 years age group. Recently, TAVR for dialysis patients was approved in Japan. The choice of SAVR or TAVR for patients with AS is determined by the valvular disease team, considering age, valve durability data, SAVR procedure risk (STS score, Euro SCORE, and Japan SCORE), TAVR procedure risk, anatomic characteristics, and frailty. Conversely, according to the ESC/EACTS guidelines [26], the age of indication for TAVR is 75 years. Similarly, the Japanese guidelines [27] specify TAVR for patients aged > 80 years and SAVR for those aged < 75 years. Nevertheless, further studies are needed to determine the indications for SAVR in dialysis patients.

One of the most important risk factors for calcification in HD patients is abnormal bone mineral metabolism, including hyperphosphatemia. In a report on healthy individuals, only serum phosphorus concentration was associated with valve calcification, and it was also involved in the calcification of aortic valves, mitral valves, and the mitral annulus; however, the usefulness of statins in preventing the development of calcification in aortic stenosis has not been demonstrated [28], [29]. However, in our study, the mortality rate was lower in patients who received therapeutic intervention for dyslipidemia. The 5-year outcome comparison showed significantly more cases of hyperlipidemia in the survival than in the non-survival group. Although there was no significant difference in preoperative blood tests, the levels of total cholesterol, triglycerides, high-density lipoprotein cholesterol, and low-density lipoprotein cholesterol were higher in the survival than in the non-survival group. In HD patients, HF is caused by ischemic heart disease, valvular disease, and non-cardiac edema due to fluid retention, anemia, or arteriovenous shunts [30]. The 5-year survival rate of dialysis patients with HF is worse at 12.5% [31].

Yamauchi et al. [19] reported that an LVEF of 30–60% was a risk factor for perioperative death. In the present study, there was no significant difference in LVEF; however, significant differences were observed in the LVDd and LVDs between the groups. Inoue et al. [32] reported that LVDd was a predictor of mortality in HD patients. It is important to note that, in this study, the HR of LVDs was 0.61 and the mortality decreased with LVDs dilatation. HD patients are in a state of continuous volume and pressure overload of the LV due to weight gain during HD, increased preload from shunting, and increased afterload from hypertension and peripheral circulatory failure. In response to excessive stress on the LV wall, cardiomyocyte hypertrophy and fibroblast proliferation occur, and the LV myocardial remodeling attempts to compensate by increasing the LV wall thickness [33]. Compared with normal morphology, the risk of cardiovascular events is higher, and the prognosis is worse for concentric hypertrophy, eccentric hypertrophy, and concentric remodeling, in that order [34]. As the burden on the LV continues, the LV begins to dilate, eventually leading to HF [35].

### 4.2 In-hospital mortality according to score

The Japan SCORE (mortality) and Japan SCORE + major complications in this study were 14.9 (SD 16.2) and 30.4 (SD 14.6), respectively. We also focused on the SOFA, APACHE II, and SAPS II scores used in the ICU. In most cases of cardiovascular surgery, the patients were intubated and admitted to the ICU; thus, their condition was evaluated after extubation. The mean SOFA score was 10.9 (SD 2.6), the APACHE II score was 24.0 (SD 6.0), and the SAPS II score was 49.9 (SD 11.1), resulting in a predicted in-hospital mortality rate of approximately 40–50% [17], [36], [37]. However, the in-hospital mortality rate in this study was 8.8% (five cases), which was similar to that in previous reports [9], [12], [21], [38]. Although there was no significant difference in the results of this study, the SOFA score is a simple tool to identify organ disorders in the respiratory system, circulatory system, central nervous system, and the liver, kidney, and coagulation system; additionally, the total score is used to determine the severity of illness. Currently, the SOFA score is widely used as a method to assess the severity of illness in the ICU. We currently use the STS and Japan SCORE as indicators of preoperative mortality and complications. However, mortality was not evaluated based on surgical and perioperative data; therefore, further research is needed on the SOFA score.

### 4.3 Postoperative risk factors for 5-year mortality

In our study, the length of ICU stay and the albumin level were strong independent risk factors for mortality. Yamauchi et al. [19] reported that postoperative risk factors for mortality included bleeding, stroke, deep sternal infection, cardiac arrest, Af, prolonged ventilation, and gastrointestinal complications. Nakatsu et al. [21] reported that the significant predictors of long-term survival were concomitant CABG and prosthesis size. In this study, the possible factors affecting the length of ICU stay were the requirement for IABP or ECMO during the postoperative period, intubation time, and complications, which were greater in the non-survival than in the survival group, but not significantly different. However, hypoalbuminemia has been shown to be an independent marker for predicting mortality in HD patients [39], [40]. Chen et al. [41] maintained the serum albumin level of HD patients at approximately 3.5 g/dL for 2 years. The 3-year survival rate was higher in the group with serum albumin levels maintained at ≥ 3.5 g/dL. The albumin level at discharge in our study was as low as 2.5 g/dL (SD 0.7 g/dL) in the non-survival group and 3.0 g/dL (SD 0.4 g/dL) in the survival group. In addition, a serum albumin level of < 3.2 g/dL increases the risk of developing frailty by 1.89 times if the patient remains undernourished [42].

### 4.4 Limitations

There were several limitations to this study. First, this was a retrospective study. Second, postoperative follow-up was difficult in HD patients; hence, sufficient echocardiographic follow-up data were unavailable. Third, the frequency of valve replacement surgery in HD patients was low, and we could not perform an adequate statistical analysis.

## 5 Conclusion

This study suggests that independent factors for 5-year mortality were age, hyperlipidemia, LVDd and LVDs, Japan SCORE, length of ICU stay, and hypoalbuminemia at discharge. Nevertheless, further studies are needed to clarify whether preoperative echocardiographic LV parameters and improvement in hypoalbuminemia at discharge can improve the 5-year prognosis of HD patients undergoing SAVR.

### CRediT authorship contribution statement

**Ikuko Shibasaki:** Formal analysis, Writing – original draft. **Taira Fukuda:** . **Hironaga Ogawa:** . **Go Tsuchiya:** . **Yusuke Takei:** . **Masahiro Seki:** . **Takashi Kato:** . **Yuta Kanazawa:** . **Shunsuke Saito:** . **Toshiyuki Kuwata:** . **Yasuyuki Yamada:** . **Yasuo Haruyama:** Formal analysis. **Hirotsugu Fukuda:** Writing – original draft.

## Declaration of Competing Interest

The authors declare that they have no known competing financial interests or personal relationships that could have appeared to influence the work reported in this paper.
